# Quantitative Magnetization Transfer Imaging as a Biomarker for Effects of Systemic Inflammation on the Brain

**DOI:** 10.1016/j.biopsych.2014.09.023

**Published:** 2015-07-01

**Authors:** Neil A. Harrison, Ella Cooper, Nicholas G. Dowell, Georgia Keramida, Valerie Voon, Hugo D. Critchley, Mara Cercignani

**Affiliations:** a Brighton and Sussex Medical School, University of Sussex, Brighton.; b Sackler Centre for Consciousness Science, University of Sussex, Falmer.; c Sussex Partnership National Health Service Trust, Brighton.; d Department of Psychiatry, University of Cambridge, Cambridge, United Kingdom.; e Cambridge and Peterborough NHS Foundation Trust, Cambridge, United Kingdom.; f Neuroimaging Laboratory, Santa Lucia Foundation, Rome, Italy.

**Keywords:** Biomarker, Cytokine, Depression, Fatigue, Inflammation, Insula, MRI

## Abstract

**Background:**

Systemic inflammation impairs brain function and is increasingly implicated in the etiology of common mental illnesses, particularly depression and Alzheimer’s disease. Immunotherapies selectively targeting proinflammatory cytokines demonstrate efficacy in a subset of patients with depression. However, efforts to identify patients most vulnerable to the central effects of inflammation are hindered by insensitivity of conventional structural magnetic resonance imaging.

**Methods:**

We used quantitative magnetization transfer (qMT) imaging, a magnetic resonance imaging technique that enables quantification of changes in brain macromolecular density, together with experimentally induced inflammation to investigate effects of systemic inflammatory challenge on human brain microstructure. Imaging with qMT was performed in 20 healthy participants after typhoid vaccination and saline control injection. An additional 20 participants underwent fluorodeoxyglucose positron emission tomography following the same inflammatory challenge.

**Results:**

The qMT data demonstrated that inflammation induced a rapid change in brain microstructure, reflected in increased magnetization exchange from free (water) to macromolecular-bound protons, within a discrete region of insular cortex implicated in representing internal physiologic states including inflammation. The functional significance of this change in insular microstructure was demonstrated by correlation with inflammation-induced fatigue and fluorodeoxyglucose positron emission tomography imaging, which revealed increased resting glucose metabolism within this region following the same inflammatory challenge.

**Conclusions:**

Together these observations highlight a novel structural biomarker of the central physiologic and behavioral effects of mild systemic inflammation. The widespread clinical availability of magnetic resonance imaging supports the viability of qMT imaging as a clinical biomarker in trials of immunotherapeutics, both to identify patients vulnerable to the effects of systemic inflammation and to monitor neurobiological responses.

Systemic inflammation impairs mood, cognition, and behavior (1). However, the importance of central actions of systemic inflammation to the etiology of common mental illnesses has been recognized only more recently (2, 3, 4). Proinflammatory cytokines, particularly interleukin-1, interleukin-6 (IL-6), and tumor necrosis factor-α, are among the principal mediators of these immune influences on the brain. These soluble factors are released by activated immune cells following pathogen exposure, and they orchestrate and integrate whole-organism responses to infection by coordinating peripheral immunologic and central behavioral reactions. Cytokines are also released in response to acute and chronic psychosocial stress (5), and elevated levels are consistently reported in people with depression (6, 7). When inflammation or stress becomes chronic, their effects can become pathologic and contribute to the development of neuropsychiatric diseases, including depression.

Clinical trials of immunotherapeutics that target proinflammatory cytokines indicate that tumor necrosis factor-α blockers may be useful in treating fatigue and other vegetative symptoms in patients with subsyndromal depression (8, 9). The same seems to be true for a proportion of patients with major depression in whom increased peripheral inflammatory markers appear to be a prerequisite for anti–tumor necrosis factor-α efficacy (10). However, marked response variability, even within inflamed individuals, suggests that for depression, vulnerability to the central effects of systemic inflammation may be more critical than levels of inflammation per se. Correspondingly, preclinical studies in both rodents and humans show that inflammation activates a neurally mediated immune-brain communicatory pathway projecting to insula cortex within hours of acute inflammatory challenge (11, 12, 13). This pathway is highly dynamic; for example, there is a fourfold increase in immune-sensitive vagus afferent neurons after reexposure to the same pathogen (14). A one-to-one mapping between peripheral and central effects of circulating inflammatory cytokines is unlikely. Positron emission tomography (PET) studies, using the peripheral benzodiazepine receptor PBR28 marker of microglial activation, reported similar variability in the sensitivity of brain microglia to lipopolysaccharide-induced inflammation in baboons (15). Interindividual differences in insula reactivity to a standard peripheral inflammatory challenge, rather than the absolute magnitude of peripheral inflammatory response, best predict behavioral effects (particularly fatigue) of inflammation in humans (13). Taken together, these data suggest that, on its own, a peripheral biomarker is unlikely to predict accurately behavioral responses to inflammation or immunotherapies. There is a critical need to identify a brain-based biomarker of the central effects of inflammation that can be rapidly adopted clinically.

Current human studies investigating the central effects of systemic inflammation largely use functional magnetic resonance imaging (MRI) measures of regional brain blood flow (13, 16, 17, 18). These functional MRI methods have played a key role in defining the network of brain regions sensitive to systemic inflammation. However, functional MRI is unable to provide detailed insight into the mechanisms underlying these effects. In addition, its dependence on long scanning sessions, performance of tasks by participants, and complex postscanning analyses constrains its utility as a clinically viable biomarker. Alternative imaging modalities, such as fluorodeoxyglucose (FDG)-PET (19, 20, 21) or PET markers of microglial activation such as PBR28 (15), provide proximate information about the mechanisms underlying inflammation-induced change in function. However, the high cost, reliance on ionizing radiation, and demanding user-dependent processing of such modalities limit their clinical utility. By contrast, conventional structural MRI techniques are limited by their almost total dependence on signals from free water components of tissue, making them effectively blind to changes in more biologically significant macromolecular tissue components.

To address these limitations, we investigated whether quantitative magnetization transfer (qMT) imaging, an advanced structural MRI technique exploiting the phenomenon of magnetization transfer (MT) between free and macromolecular bound protons, is sensitive to the effects of systemic inflammation in humans. In the rodent, MT imaging has demonstrated sensitivity to the effects of peripheral inflammation on the brain (22) and sciatic nerve (23). In the present study, we induced mild systemic inflammation in human volunteers using typhoid vaccination, a validated model of inflammation that engenders an approximately threefold increase in circulating proinflammatory cytokines (16), consistent with the magnitude of inflammation observed in patients with depression (6).

In the first study, 20 healthy participants underwent qMT imaging twice, once after random administration of typhoid vaccine and once after placebo (saline) injection. Based on the preclinical literature using naturalistic challenges such as inhaled antigens (24, 25), typhoid vaccination (13), and lipopolysaccharide injection (26), we predicted an inflammation-induced increase in magnetization exchange between free and macromolecular bound protons within the insula, a region implicated in representing bodily physiologic state across physiologic domains including inflammation (27, 28) and their translation into subjective experiences such as warmth, hunger, or fatigue (29, 30). We included additional basal ganglia regions of interest based on their reported sensitivity to inflammation induced by repeat injections of the cytokine interferon-α (19, 31).

To investigate the neurobiological basis of our observed changes in qMT further, we performed a second study undertaking a fresh analysis of FDG-PET data previously acquired to investigate the effects of inflammation on memory function (21). In this study, a second cohort of 20 healthy control subjects underwent FDG-PET imaging before and after both typhoid vaccination and saline injection. We hypothesized that increases in MT within the insula would reflect localized increases in glial-neuronal metabolic interactions and glucose uptake within this region (32).

## Methods and materials

### Participants

For the qMT study, 20 healthy nonsmokers (7 men; mean age, 26.4 ± 6.4 years) were recruited; for the PET study, 20 additional healthy male nonsmokers (mean age, 24.7 ± 6.8 years) were recruited. All participants were screened for relevant physical or psychiatric illness. Volunteers who had received typhoid vaccine within 3 years or other vaccine within 6 months were excluded. All participants were advised not to consume caffeinated beverages or alcohol, to avoid high-fat meals, and to refrain from excessive exercise for 24 hours before testing. They were also asked not to take antiinflammatories or antibiotics for 14 days before testing. All participants were medication-free. Written informed consent was obtained after complete description of the study to the participants. Study procedures were approved by the Hertfordshire (qMT study) and Brighton East (PET study) National Research Ethics Committees.

### Study Design

We adopted randomized, double-blind, crossover repeated measures designs for both studies. In the qMT study, all participants underwent two separate scanning sessions conducted at least 7 days apart. In the first session, participants were randomly assigned to one of two experimental conditions (typhoid vaccine or placebo saline injection). Both participant and researcher (EC) were blind to treatment allocation. In the first session, 10 participants received typhoid vaccination (Typhim Vi; Aventis Pasteur MSD, Maidenhead, Berkshire, United Kingdom), and 10 participants received placebo injection. The Salmonella typhi vaccine experimental model of inflammation induces a low-grade inflammatory response associated with an approximately threefold increase in peripheral IL-6 levels peaking 3–4 hours after vaccination (16). Baseline blood sample, blood pressure, and temperature were taken first followed by injections of .025 mg S. typhi or .5 mL normal saline. Imaging was performed 3–4 hours after injection in a 60-min scanning session. During each MRI scanning session, we acquired qMT, structural imaging data, and functional MRI data at rest and during a prospective memory task (not reported here). A second blood sample was taken 4 hours after injection. Body temperature, blood pressure, and pulse were assessed at baseline and 4 hours using an aural digital thermometer (Braun ThermoScan; Braun GmbH, Kronberg, Germany) and automated digital blood pressure monitor (OMRON M6 Comfort; OMRON Healthcare, Kronberg, Germany) as appropriate. The second MRI scanning session was identical to the first except that participants received the alternate injection (i.e., typhoid vaccination if they previously received saline and vice versa).

In the PET study, each participant underwent three consecutive resting FDG-PET imaging sessions each separated by 4 hours. After the first scanning session, 13 randomly selected participants received an intramuscular injection of .025 mg S. typhi vaccine, and 7 received .5 mL normal saline administered intramuscularly into the deltoid muscle. After the second PET scan, the injections were reversed (i.e., 13 participants received the saline injection, and 7 received S. typhi vaccine). This design enabled us to measure effects of typhoid vaccine on regional resting brain glucose metabolism, while controlling for nonspecific effects of time. Body temperature, blood pressure, pulse, and blood samples for cytokine measurement were taken before each PET scan. Subjective sickness symptoms, including fatigue, were monitored every 3 hours using the Profile of Mood States (POMS) questionnaire.

### Cytokine Analyses

Blood (20 mL) was drawn into BD Vacutainer tubes (Becton, Dickinson and Company, Franklin Lakes, New Jersey) containing ethylenediamine tetraacetic acid (EDTA) anticoagulant and centrifuged immediately at 1250*g* for 10 min. Plasma was removed, aliquoted, and frozen at −80°C before analysis. Plasma IL-6 was assessed using high-sensitivity enzyme-linked immunosorbent assays (R&D Systems, Abingdon, United Kingdom). The limit of detection of the IL-6 assay is .039 pg/mL, with intra-assay and interassay coefficients of variation of 7.4% and 7.8%, respectively. Cytokine analysis was performed using mixed measures analyses of variance and subsequent paired sample *t* tests in IBM SPSS Statistics version 21.0 (IBM Corp, Armonk, New York).

### Behavioral Analyses

In the qMT study, the effects of inflammation on mood and fatigue were measured using a fatigue visual analog scale and the POMS; in the PET study, only POMS data were acquired. The Beck Depression Inventory and the Spielberger State and Trait Anxiety Inventory were completed to index baseline levels of depression and anxiety symptoms, respectively. Behavioral analysis was performed in IBM SPSS Statistics version 21.0 using mixed measures analyses of variance and subsequent paired sample *t* tests.

### Image Acquisition

All MRI was performed on a 1.5-tesla Siemens Avanto (Siemens AG Medical Solutions, Erlangen, Germany) equipped with a 32-channel head-coil and maximum gradient strength of 44 mT/m. The qMT protocol was based on the balanced steady-state free precession method (33). The acquisition was performed using a three-dimensional True Fast Imaging with Steady-state Precession sequence (field of view = 240 mm × 180 mm, matrix = 256 × 96, slices = 32, slice thickness = 5 mm), modified to allow the duration of the radiofrequency pulse to be varied. There were 22 volumes acquired varying either the flip angle (between 5° and 40°) or the repetition time (between 3.66 msec and 5.96 msec) and the pulse duration. T1 mapping was performed by acquiring three three-dimensional fast low-angle shot volumes with excitation flip angles 5°, 15°, 25°; repetition time = 30 msec and echo time = 5 msec; and same field of view, matrix, and number of slices as the True Fast Imaging with Steady-state Precession. The total scan time for the MT and T1 mapping data was ~ 8 min. A three-dimensional T1-weighted anatomic scan was obtained for each participant in one session using a magnetization prepared rapid acquisition gradient-echo acquisition (repetition time = 2730 msec, echo time = 3.57 msec, inversion time = 1000 msec, flip angle = 7°).

### Image Analysis

The MT and T1 mapping data from both sessions were first realigned to subject-specific magnetization prepared rapid acquisition gradient-echo structural images using the SPM8 rigid-body registration function (Wellcome Trust Centre for Neuroimaging, University College London, UK; http://www.fil.ion.ucl.ac.uk/spm). Magnetization prepared rapid acquisition gradient-echo images were then segmented into white and gray matter and cerebrospinal fluid using the SPM8 “new segment” function to yield a parenchymal mask. A T1 map was calculated for all data sets by fitting the theoretical spoiled gradient echo as a function of the flip angle to the signal measured by the three-dimensional fast low-angle shot sequences (34). The MT parameters were obtained by performing a voxel-wise nonlinear least-squares fitting (Levenberg-Marquardt method) to a binary spin bath model for balanced steady-state free precession (33); this yields bound proton fraction, MT exchange rate constant (k_f_), and T2 of the free water component. The quantitative maps were masked to remove background noise, warped into standard Montreal Neurological Institute space using the segmentation deformation fields, and then smoothed using an 8-mm^3^ full-width at half maximum Gaussian kernel. Paired sample *t* tests were used to estimate the effect of typhoid vaccine–induced inflammation on the qMT parametric maps, and regression analysis was used to investigate relationships between changes in qMT parameters and fatigue measured on the fatigue visual analog scale.

### Regions of Interest

Region of interest masks for the whole of the left and right insular cortex and basal ganglia (encompassing the caudate nucleus, putamen, globus pallidus, and nucleus accumbens) were created using the WFU PickAtlas (http://fmri.wfubmc.edu/software/PickAtlas). Results are reported for clusters surviving family-wise error correction at *p* < .05 for each region of interest. Additional “secondary” regions of interest were produced in a similar manner for whole-brain regions (bilateral frontal, parietal, temporal, and occipital cortex; thalamus; and cerebellum) and discrete regions previously implicated in mediating behavioral responses to inflammation (bilateral amygdala, hippocampus, caudate, putamen, ventral striatum (35), substantia nigra, and Brodmann area 25). Mean change in k_f_ for all voxels within the mask were calculated using MarsBar (36) and are reported in Supplement 1.

### Multiple Comparisons

We conducted a Monte Carlo simulation with 1000 iterations using software written in MATLAB (The Mathworks, Natick, MA; https://www2.bc.edu/~slotnics/scripts.htm) (37) to correct for multiple comparisons across the whole brain at a corrected significance level of *p* < .05. Only clusters of activation >78 contiguous voxels outside our predefined regions of interest are reported.

### PET Imaging

All PET scans (mean, 155.3 ± 11.8 MBq FDG) were acquired for 35 min on a Siemens Biograph-64 PET-CT scanner (Siemens AG Medical Solutions) and analyzed in SPM8. Images were corrected for scatter, random effects, and effects of attenuation and then reconstructed in 1-min windows using Siemens proprietary iterative three-dimensional reconstruction schema (21 iterations and 8 subsets). Individual 1-min scans were realigned and summed to produce a single 35-min activation scan per session. This scan was coregistered to subjects’ structural MRI scans and then spatially smoothed with an 8-mm^3^ full-width at half maximum Gaussian kernel. Main effects of inflammation on resting brain glucose metabolism were determined by comparing normalized activation scans at baseline and 4 hours after vaccination within a paired sample *t* test. Exclusive masking with activation scans at 4 hours minus baseline after placebo (threshold *p* < .005) was used to account for nonspecific effects of time. Normalization to a grand mean scaled value of 50 mL/100 g/min was applied, and global effects were included as nuisance covariates in the general linear model (analysis of covariance). Changes in FDG uptake for the peak voxel within predefined regions of interest were extracted and regressed against changes in subjective fatigue (POMS tiredness subscale) in IBM SPSS Statistics version 21.0.

## Results

### Inflammatory Cytokine Response to Vaccination

**Figure 1:**
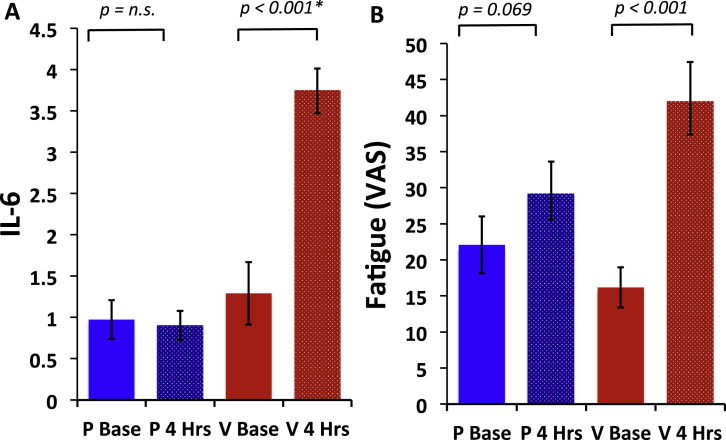
Effects of inflammation on interleukin-6 and fatigue. **(A)** Change in circulating interleukin-6 before and after vaccine (V base and V 4 Hrs) and placebo injection (P base and P 4 Hrs). Typhoid vaccination evoked a robust inflammatory response with an approximately threefold increase in plasma interleukin-6 from mean (± SE) 1.29 ± .38 pmol/L at baseline to 3.74 ± .27 pmol/L at 4 hours [*t*_19_ = 5.93, *p* < .001]. The placebo condition was not associated with any change in interleukin-6 from .97 ± .23 pmol/L at baseline to .90 ± .18 pmol/L at 4 hours [*t*_19_ = −.54, *p* = .59]. **(B)** Change in fatigue before and after typhoid vaccination and placebo saline injection. Typhoid vaccination, but not placebo injection, was associated with a significant increase in fatigue as shown by mean (± SE) fatigue visual analog scale 16.18 ± 12.57 at baseline to 42.40 ± 22.52 at 4 hours [*t*_19_ = 6.05, *p* < .001] for typhoid vaccination and 22.08 ± 17.72 at baseline to 29.60 ± 17.94 at 4 hours [*t*_19_ = 1.93, *p* = .07] for placebo injection. IL-6, interleukin-6; *n.s., p* = non significant; VAS, visual analog scale.

**Figure 2:**
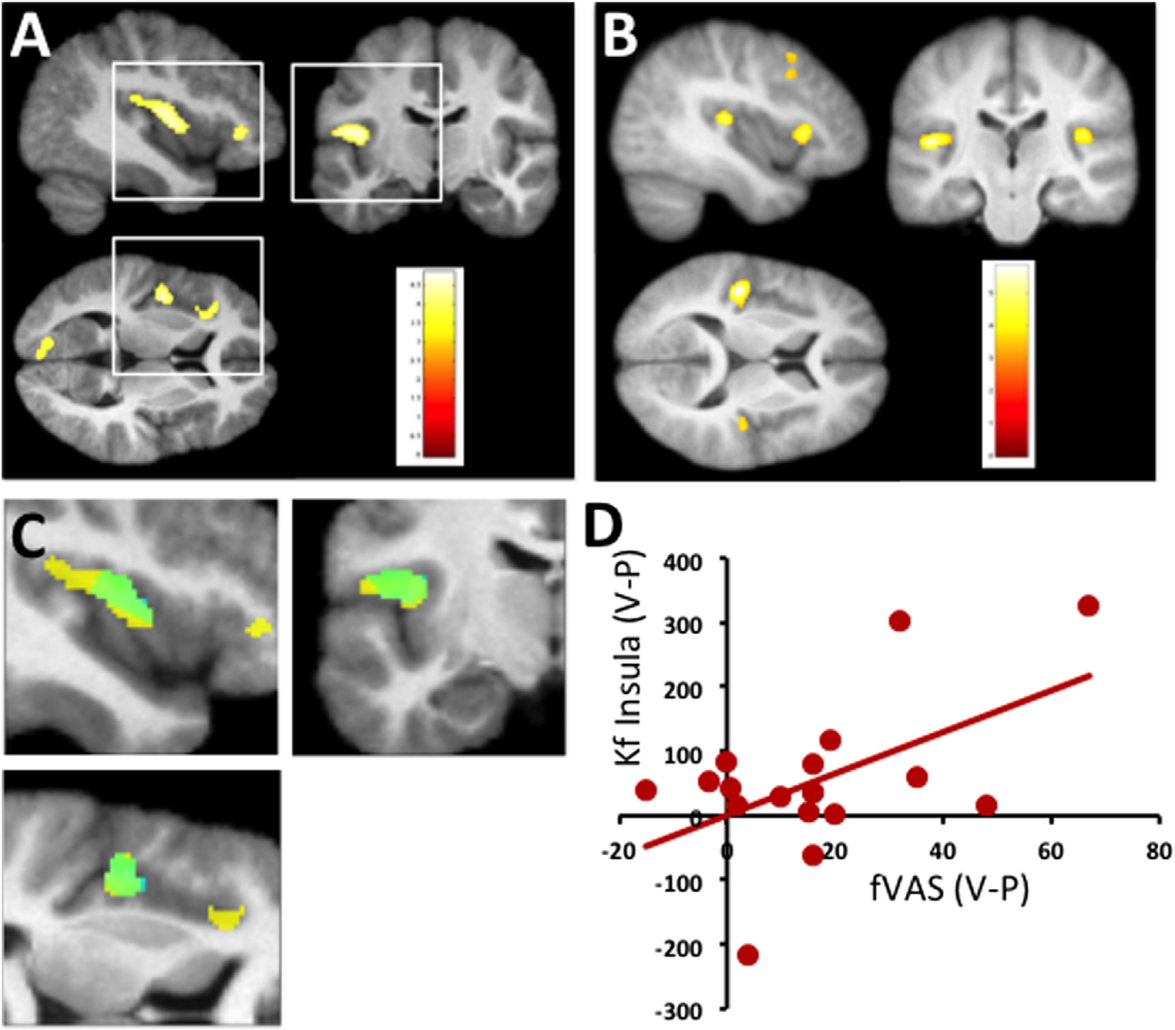
Effects of inflammation on brain structure and function. **(A)** Quantitative magnetization transfer imaging study. Brain regions showing a significant increase in magnetization transfer exchange rate constant 3–4 hours after typhoid vaccine–induced inflammation compared with control (saline) injection. Data displayed at a whole-brain corrected threshold of *p* < .05. Color scale denotes *t* score. **(B)** Fluorodeoxyglucose positron emission tomography imaging study. Brain regions showing a significant increase in fluorodeoxyglucose uptake 3–4 hours after typhoid vaccine–induced inflammation; exclusively masked by changes in fluorodeoxyglucose uptake 3–4 hours after placebo (mask threshold *p* < .005). Data displayed at a whole-brain corrected threshold of *p* < .05. **(C)** Left insula voxels showing a significant increase in magnetization transfer exchange rate constant 3–4 hours after experimentally induced inflammation (yellow) overlaid with voxels (green) additionally predicting inflammation-induced fatigue (fatigue visual analog scale, *p* < .05). **(D)** Correlation of fatigue visual analog scale scores 4 hours after typhoid vaccine minus placebo (V − P) (x-axis) with inflammation-induced changes in magnetization transfer exchange rate constant of all 1196 voxels within the posterior insula cluster (illustrated in yellow in C) on the y-axis (*R*^*2*^ = .2, *p* < .05). fVAS, fatigue visual analog scale; k_f_, magnetization transfer exchange rate constant.

Typhoid vaccination evoked an approximately threefold increase in plasma IL-6 from mean (±SE) 1.29 ± .38 pmol/L at baseline to 3.74 ± .27 pmol/L at 4 hours [*t*_19_ = 5.93, *p* < .001] (Figure 1A). The placebo condition was not associated with any change in IL-6 (.97 ± .23 pmol/L at baseline to .90 ± .18 pmol/L at 4 hours) [*t*_19_ = −.54, *p* = .59] (Figure 2A). This finding was confirmed by a significant treatment by time interaction [*F*_1,19_ = 34.79, *p* < .001]. A similar increase in IL-6 after typhoid vaccination was also observed in the PET imaging cohort (from 1.78 ± 1.09 pmol/L before vaccination to 4.48 ± 2.24 pmol/L 4 hours after vaccination) [*t*_19_ = 5.85, *p* < .001]. No change in body temperature was observed in either sample (*p* > .4).

### Psychological Effects of Inflammation

Consistent with previous reports (13), typhoid vaccination, but not placebo, was associated with a significant increase in reported fatigue: fatigue visual analog scale 16.18 ± 12.57 (baseline) to 42.40 ± 22.52 (4 hours) [*t*_19_ = 6.05, *p* < .001] for typhoid vaccination and 22.08 ± 17.72 (baseline) to 29.60 ± 17.94 (4 hours) [*t*_19_ = 1.93, *p* = .07] for placebo injection (Figure 2B). This association was confirmed by a significant treatment by time interaction [*F*_1,19_ = 11.94, *p* < .003]. A similar increase in fatigue after typhoid vaccination was also observed in the PET imaging cohort with POMS tiredness subscale scores increasing from 8.75 ± 2.45 at baseline to a peak of 13.85 ± 4.27 after typhoid vaccination [*t*_19_ = 6.83, *p* < .001].

### Effects of Acute Inflammation on qMT

Inflammatory challenge was associated with a significant increase in k_f_ within six predominantly left-sided regions each surviving a whole-brain corrected threshold of *p* < .05 (Table 1). Approximately 50% of these voxels (Figure 1A) were located within posterior insula or anterior insula, regions previously shown to be functionally sensitive to systemic inflammation (13). Preplanned region-of-interest analyses confirmed that both the posterior insula and the anterior insula clusters survived stringent family-wise error correction at *p* < .05 (Table 1). No significant changes were observed in the right insula or the basal ganglia regions of interest at this threshold, and no changes were observed for the subsidiary MT parameters bound proton fraction or T2 of the free water component. Further inspection of the insular clusters demonstrating inflammation-induced changes in k_f_ showed that most left posterior insular voxels additionally predicted inflammation-induced changes in fatigue (*p* < .05) and together explained ~ 24% of the variance (green in Figure 1C,D). We observed no significant correlation between the left insula region showing a main effect of inflammation and IL-6 (*p* = .16, *R*^*2*^ = .06) or between IL-6 and fatigue (*p* = .13, *R*^*2*^ = .08).

### Effects of Inflammation on Resting Glucose Metabolism

Finally, we undertook a fresh examination of a FDG-PET data set (from an independent sample of healthy individuals) to investigate whether our observed changes in k_f_ were related to metabolic changes in glucose metabolism. This analysis demonstrated significant increases in glucose metabolism within a discrete network of regions centered on the insula with additional changes within prefrontal cortices. Preplanned region-of-interest analyses confirmed this finding with left posterior insula (but not right posterior or left anterior insular clusters) surviving stringent family-wise error correction at *p* < .05 (Table 2). Changes in FDG uptake within the left posterior insula explained ~ 20% of the variance in fatigue after inflammation (*R*^*2*^ = .20, *p* = .048). No significant changes in glucose metabolism were observed within either basal ganglia region of interest.

## Discussion

Our data demonstrate that qMT, a structural MRI technique that is easily implemented, is sensitive to the effects of inflammation on brain molecular structure in regions known to be functionally sensitive to inflammation. Specifically, systemic inflammation resulted in an increased rate of MT from free (water) to macromolecular-bound protons within discrete left posterior and anterior insular regions. This shift in MT is consistent with an inflammation-induced increase in hydrophilic macromolecules (38) within the terminal cortical projection of this key immune-brain communicatory pathway. The functional significance of this change in insular microstructure was supported further by our between-subject analysis that demonstrated a correlation with individual susceptibility to inflammation-induced fatigue, one of the earliest and most commonly observed consequences of inflammation and a key symptom of depression (13, 39). Our FDG-PET data demonstrated a similar insula-focused increase in glucose metabolism within 4 hours of inflammatory challenge. Together, these data demonstrate that qMT is not only sensitive to the central effects of systemic inflammation but also can differentiate individuals who are most susceptible to the motivationally impairing effects of inflammation, critical features of a biomarker with clinical utility.

Structural MRI techniques typically exploit gross differences in T1 and T2 relaxation of water molecules to produce image contrast. However, within biological tissue, protons exist in both free (water) and molecule-bound environments, each with its own distinct T1 and T2 properties. Traditional structural MRI techniques are insensitive to these differences in molecular environment. In contrast, computational modeling of MT weighted images enables quantification of the rate of magnetization exchange (k_f_) at this critical interface providing an indirect quantification of microstructural changes in protein, lipid, and hydrophilic macromolecular density across the whole brain. Although myelin dominates this MT exchange process in central nervous system white matter (40, 41), other processes such as inflammation (32, 33) and metabolic (42) and pH changes (33, 41, 43) appear to play an important role in other neuronal structures. Hydrophilic macromolecules rich in hydroxyl, amine, and carboxyl groups appear to be particularly important to this process (38).

Our finding of a localized increase in insula k_f_ may relate to targeted cytokine release from astrocytes or neurons or both (44, 45, 46, 47). However, the associated changes in glucose uptake (FDG-PET) suggest that this increase in insula k_f_ more likely relates to a localized increase in metabolically active macromolecules such as lactate. This model is supported further by the relatively small increases in cytokine gene expression typically observed after activation of this immune-brain communicatory pathway (47). Historically, increased FDG uptake has been linked to increased neuronal metabolism. However, investigations suggest that astrocytes may account for 50% of all brain glucose uptake, even at rest (32, 48). Following glutamatergic activity, there is a further shift of glucose use away from neurons toward astrocytes (49) associated with a sustained increase in extracellular lactate release from astrocytes (50). Lactate, which is present in high concentrations under these conditions (51, 52), has a hydroxyl group adjacent to the carboxyl group, chemical structures that heavily influence MT (38). A relationship between k_f_ and FDG uptake has previously been suggested in Alzheimer’s disease, where reductions in k_f_ (referred to as RM_0_^B^) were reported in patients within cortical regions associated with reduced FDG uptake (42).

In humans, studies inducing inflammation with “naturalistic” challenges, such as inhaled antigens (24, 25), typhoid vaccination (13), and lipopolysaccharide (26), as well as acute psychosocial stressors (53) consistently identify insular cortex as a key neural substrate for the central representation of inflammation. Many of these studies additionally demonstrate correlations between inflammation-induced changes in insular function and behavioral effects, including fatigue (13) and experience of social exclusion pain (53). These studies are noteworthy because they accord with a broader literature demonstrating a central role for the insula in providing a cortical representation of the physiologic state of the body across physiologic domains (27, 28, 29) and the translation of this information into subjectively experienced feeling states (e.g., warmth, hunger, fatigue). However, previously reported effects of peripheral inflammation on brain function are not limited to the insula. Task-dependent functional changes corresponding to discrete effects on mood, cognition, and motivation have been previously described for brain regions including frontal cortex, subgenual cingulate, ventral striatum, amygdala, hippocampus, and substantia nigra. Secondary exploratory analysis (reported in Supplement 1) reveals inflammation-induced changes in k_f_ in many of these regions (although only the insula survives Bonferroni correction). Future studies will need to determine the importance of these exploratory findings.

Studies inducing inflammation with supraphysiologic doses of interferon-α are associated not with an increase in insular activity, but rather well-localized changes in basal ganglia function (19). These changes include bilateral (although left predominant) increases in FDG uptake after 4 weeks of interferon-α treatment; correlation of left basal ganglia FDG uptake changes with interferon-α-induced fatigue (19); and left, but not right, sided increases in basal ganglia glutamate-to-creatine ratio (measured using magnetic resonance spectroscopy), which again correlated with interferon-α-induced fatigue measured as a reduction in motivation (31). In the present analysis, we did not detect any change in k_f_ or FDG-glucose uptake in either the left or the right basal ganglia regions of interest at the stringent thresholds adopted. However, similar to the studies using high-dose interferon-α inflammatory challenges, typhoid vaccine–induced inflammation evoked changes in k_f_ that were markedly left-lateralized.

In the present study, we used FDG-PET rather than a more specific marker of microglial activation such as PK11195 to index the effects of inflammation on resting brain function. Although this approach enabled us to detect functional changes resulting from metabolic activation of any immune-brain communicatory pathway (e.g., activation of vagal afferent nerve traffic, transport of cytokines across the blood-brain barrier, release of prostaglandins by endothelial cells, and activation of microglia particularly within the circumventricular organs) (28), it did not allow us to determine whether these changes were mediated by microglial activation. Future studies will need to determine whether microglial activation demonstrated after potent inflammatory challenges (15) is similarly observed after mild systemic inflammation comparable to that observed in depression (54).

Other limitations of our study include ethical constraints that mandated use of an all-male cohort in our PET study. Although we have no evidence to support sex-specific effects of the typhoid vaccine experimental model, this should be considered in the extrapolation of our data. Similarly, the qMT and PET data were acquired from two independent samples. From a technical perspective, the qMT and T1 mapping data were collected without a B1 map. However, B1 inhomogeneity is small at a B0 field strength of 1.5 Tesla using radiofrequency transmission from a body coil (55). In addition, our imaging data were acquired with low flip angle pulses (maximum 40°) minimizing B1 field imperfections further. Although we do not believe B1 mapping was necessary for this study, it may be more important to consider when using balanced steady-state free precession qMT at higher field strengths.

In conclusion, to date conventional neuroimaging techniques have been too insensitive, cumbersome, or expensive to function as viable clinical biomarkers for studies investigating the efficacy of immunotherapies. We demonstrate that qMT is sensitive to the central effects of systemic inflammation and that the technique is able to differentiate individuals most sensitive to the behavioral effects of systemic inflammation. Our findings indicate the viability of qMT imaging for clinical biomarking in trials of immunotherapeutics and studies of neuropathology.

## Figures and Tables

**Table 1:** Main Effect of Inflammation: Vaccine > Placebo on k_f_ (Whole-Brain) Threshold *p* < .001

Side	Region	Coordinates	*Z* Score	k	Uncorrected *p*	Corrected *p*
L	Posterior insula	−52 −18 15	3.79	1196	<.001	.008^a^
L/R	Precuneus	−6 −57 15	3.62	407	<.001	NA
L	Inferior parietal lobe	−57 −64 31	3.47	470	<.001	NA
L	Anterior insula	−39 30 0	3.45	290	<.001	.043^a^
L	TPJ	−57 −43 21	3.40	502	<.001	NA
L	Striate cortex	−10 −78 12	3.39	416	<.001	NA

k_f_, magnetization transfer exchange rate constant; L, left; NA, not applicable; R, right; TPJ, temporoparietal junction

a Pre-planned region of interest.

**Table 2:** Main Effect of Inflammation on Resting FDG Metabolism 4 Hrs−Baseline (Vaccine) Masked by 4 Hrs–Baseline (Placebo)

Side	Region	Coordinates	*Z* Score	k	Uncorrected *p*	Corrected *p* Peak
L	Posterior insula	−45 −24 10	4.88	384	<.001	.015^a^
L	Anterior insula	−36 21 3	4.26	283	<.001	.157
R	Posterior insula	40 −22 13	4.09	181	<.001	.274
R	Midfrontal gyrus	30 −7 52	4.05	157	<.001	.304
L	Midfrontal gyrus	−42 15 39	3.58	296	<.001	.800
R	Midfrontal gyrus	46 9 31	3.37	81	<.001	.945

FDG, fluorodeoxyglucose; L, left; R, right.

a Pre-planned region of interest

## Footnotes

Appendix A Supplementary material cited in this article is available online at doi:10.1016/j.biopsych.2014.09.023.
