# Supplementary material for: Quantitative Magnetization Transfer Imaging as a Biomarker for Effects of Systemic Inflammation on the Brain
Source: Biol Psychiatry. 2015 Jul 1;78(1):49–57. doi: 10.1016/j.biopsych.2014.09.023 (PMC4503794; doi:10.1016/j.biopsych.2014.09.023)
Supplement: Supplementary file 1 — Supplementary Material [file mmc1.pdf]

Quantitative Magnetization Transfer Imaging as a Biomarker for Effects of Systemic Inflammation on the Brain

Supplementary Information

Data in Tables S1 and S2 report mean change in kf for all voxels within secondary regions of interest. Table S1 reports findings for large brain regions following the approach adopted by Hannestad *et al.* (1). Table S2 reports findings for other regions previously reported to show effects of inflammation on task-dependent activity. The ventral striatal region of interest used was that reported in Martinez *et al.* (2).

All data are reported uncorrected for multiple comparisons. As such they are highly permissive and included only to inform future studies. Of note, the only region demonstrating a significant increase in kf that survives Bonferroni correction for the 28 secondary regions of interest is the left insula as reported in the main manuscript.

Supplemental References

1. Hannestad J, DellaGioia N, Gallezot JD, Lim K, Nabulsi N, Esterlis I, *et al.* (2013): The neuroinflammation marker translocator protein is not elevated in individuals with mild-to-moderate depression: A [^{11}C]PBR28 PET study. *Brain Behav Immun* 33:131-8

2. Martinez D, Slifstein M, Broft A, Mawlawi O, Hwang DR, Huang Y (2003): Imaging human mesolimbic dopamine transmission with positron emission tomography, part II: amphetamine-induced dopamine release in the functional subdivisions of the striatum. *J Cereb Blood Flow Metab* 23:285–300.

Table S1. Mean change in kf for all voxels within secondary regions of interest, following the approach adopted by Hannestad *et al.* (1).

Side	Region	*p* Value
L	Frontal Cortex	0.0078
R	Frontal Cortex	0.018
L	Temporal Cortex	0.011
R	Temporal Cortex	0.038
L	Parietal Cortex	0.0077
R	Parietal Cortex	0.024
L	Occipital Cortex	0.0052
R	Occipital Cortex	0.051
**L**	**Insula**	**0.00041***
**R**	**Insula**	**0.024**
L	Cerebellum	0.608
R	Cerebellum	0.675
L	Thalamus	0.061
R	Thalamus	0.079

Bold indicates the bilateral insula primary regions of interest.
* indicates survives Bonferroni correction for 28 secondary regions of interest.

Table S2. Mean change in kf for all voxels within secondary regions of interest. Includes regions previously reported to show effects of inflammation on task-dependent activity.

Side	Region	*p* Value
L	Amygdala	0.106
R	Amygdala	0.0092
L	Hippocampus	0.027
R	Hippocampus	0.075
L	BA25	0.412
R	BA25	0.424
**L**	**Ventral Striatum**	**0.312**
**R**	**Ventral Striatum**	**0.115**
**L**	**Putamen**	**0.029**
**R**	**Putamen**	**0.024**
**L**	**Caudate**	**0.091**
**R**	**Caudate**	**0.032**
L	Substantia Nigra	0.026
R	Substantia Nigra	0.039

Bolded values indicate basal ganglia primary regions of interest.
